# From Schools to Scans: A Neuroeducational Approach to Comorbid Math and Reading Disabilities

**DOI:** 10.3389/fpubh.2020.00469

**Published:** 2020-10-22

**Authors:** Jeremy G. Grant, Linda S. Siegel, Amedeo D'Angiulli

**Affiliations:** ^1^Department of Neuroscience, Carleton University, Ottawa, ON, Canada; ^2^Department of Educational and Counselling Psychology, and Special Education, The University of British Columbia, Vancouver, BC, Canada

**Keywords:** reading and mathematical disability, comorbidity, dyslexia, dyscalculia, psychoeducational testing, neuroimaging, developmental learning disabilities

## Abstract

We bridge two analogous concepts of comorbidity, dyslexia-dyscalculia and reading-mathematical disabilities, in neuroscience and education, respectively. We assessed the cognitive profiles of 360 individuals (mean age 25.79 ± 13.65) with disability in reading alone (RD group), mathematics alone (MD group) and both (comorbidity: MDRD group), with tests widely used in both psychoeducational and neuropsychological batteries. As expected, the MDRD group exhibited reading deficits like those shown by the RD group. The former group also exhibited deficits in quantitative reasoning like those shown by the MD group. However, other deficits related to verbal working memory and semantic memory were exclusive to the MDRD group. These findings were independent of gender, age, or socioeconomic and demographic factors. Through a systematic exhaustive review of clinical neuroimaging literature, we mapped the resulting cognitive profiles to correspondingly plausible neuroanatomical substrates of dyslexia and dyscalculia. In our resulting “probing” model, the complex set of domain-specific and domain-general impairments shown in the comorbidity of reading and mathematical disabilities are hypothesized as being related to atypical development of the left angular gyrus. The present neuroeducational approach bridges a long-standing transdisciplinary divide and contributes a step further toward improved early prediction, teaching and interventions for children and adults with combined reading and math disabilities.

## Introduction

The classification, diagnosis, and treatment of learning disabilities are important topics of research in both psychoeducational and neuroscience literature. Researchers in these two fields often measure similar constructs but use differing approaches to work with individuals with learning disabilities. Consequently, each field has produced different concepts and theories over time, leading to a sort of disconnect between the identification of learning disabilities in educational settings and the identification of learning disabilities based on neuroscientific evidence, respectively. Furthermore, the identification and development of comorbid learning disabilities, while a prevalent topic in psychoeducational literature, remains relatively understudied in neuroscience; a testable model of the neuroanatomical substrates of comorbidity is greatly needed. We developed a novel neuroeducational approach to bridge the corresponding concepts on learning disabilities in the two disciplinary fields.

### Learning Disabilities

Learning disabilities are a type of neurodevelopmental disorders that impede the acquisition, retention, or application of verbal or non-verbal information, affecting a person's ability to use specific cognitive skills (1, 2). The most prevalent learning disability is *reading disability*, a specific difficulty in learning to read, interpret, and manipulate written words, also known as *dyslexia*. The second most prevalent is *mathematical disability*, a specific difficulty in learning arithmetic and performing mental calculations, also known as *dyscalculia* (3, 4).

Current research on comorbid math and reading disabilities and their developmental origins is far from exhaustive. As recently as 2007, a systematic review of the U.S. Department of Education's Educational Research Information Center (ERIC) database revealed that the number of published studies on reading disability outnumbered the number of studies on math disability by a ratio of 14 to 1 (5). This disparity in knowledge translates into disproportionate diagnoses and asymmetric interventions for individuals with comorbid math and reading disabilities. Therefore, defining a robust neuroeducational model of dyslexia-dyscalculia comorbidity is a priority for the early identification and treatment of learning disabilities (6–8).

### The Psychoeducational Approach to Identifying Learning Disabilities

The psychoeducational evaluation is the traditional method of classifying and identifying learning disabilities. The goal of the evaluation is to examine the student's performance on standardized tests of general academic achievement (9), and will determine if the student qualifies for special education or remedial training (10). A psychoeducational assessment typically consists in obtaining an IQ score and selected domain-specific standardized tests—psychometric measures that directly assess abilities in reading, writing, or arithmetic (8, 11).

In this approach, the IQ-achievement discrepancy criterion provides the framework for identifying an unexpected difficulty with learning. To be classified as having a learning disability, the discrepancy model requires that there is a significant discrepancy (usually 1.5 standard deviations) between the person's academic ability or potential (defined by the IQ score) and academic achievement (as defined by their scores on a general reading or math test). This model rests on the questionable assumption that intelligence tests are not confounded by more basic processes for which domain-specific psychoeducational tests provide independent measures (12), and regrettably exclude the possibility of identifying learning disabilities in people with intellectual disabilities (13).

Notably, a study by Tanaka et al. (14) reported evidence based on brain activity demonstrating the diagnostic inappropriateness of the IQ discrepancy criterion. Replicating previous findings based on psychoeducational tests (15) they showed that brain activity and structures associated with reading difficulties in individuals with intact general intellectual ability and in individuals with lower intellectual ability show very similar profiles. The latter supports the recent removal of the IQ discrepancy from the definition of specific learning difficulties in the DSM-V.

### The Neuropsychological Approach to Identifying Learning Disabilities

Compared to psychoeducational evaluations, neuropsychological assessments are greater in the depth of their assessment. They are more fine-tuned to examine specific cognitive deficits (such a phonological processing deficits) that underlie learning disabilities. A neuropsychological assessment is performed by licensed clinical neuropsychologists who combine elements of brain anatomy, cognitive neuroscience, and neurodevelopment to infer the neurological correlates of differences in specific cognitive abilities (7).

Secondly, neuropsychological assessments are greater in the breadth of the assessment. In contrast to a psychoeducational evaluation (which typically consists of an IQ score and a few standardized tests), a full neuropsychological assessment includes a structured clinical interview with the client (and interviews with the client's family and/or significant others, if possible), a review of the client's relevant medical records, and the administration of tests that measure domain-general functions such as selective attention, sensory perception, fine motor skills, visuospatial reasoning, and working memory (7). All the available information will be used to make a specific diagnosis of the client's learning disability, instead of relying on psychometric measures alone.

Third, the interpretation of test scores from a neuropsychological assessment is guided by different principles than in a psychoeducational evaluation. Instead of applying the IQ-discrepancy model as in the psychoeducational approach, clinical neuropsychologists define the severity of a learning disability by introducing a cut-off threshold on the tail end of a distribution of academic achievement (9). While cut-off points are useful for providing a post-assessment diagnosis, given the limits of current causal models, they do not precisely reflect the neurobiological basis of a learning disability (13, 16); they rather emphasize normativity and address pragmatic issues related to early intervention.

The identification of math and reading disabilities is a predominant topic in *neuroeducation*—an emerging field at the intersection of neuropsychology, neuroscience, and psychoeducational research (17–19). The goals of neuroeducation are to develop curricula and teaching methods that are based on a scientific understanding of neural mechanisms of learning (20, 21). In line with this paradigm, the central theme of the present paper is linking in the most direct way possible corresponding constructs in education and neuroscience. The aim is to improve prediction of the biological and psychological factors that yield poor academic outcomes seen in individuals with learning disabilities. By examining the cognitive profiles of individuals with dyslexia and dyscalculia and mapping the observed deficits to their neuroanatomical correlates from existing research, the neuroeducational model proposed in the current research provides educators and neuroscience researchers with a working framework for designing effective teaching and interventions specific to individuals with comorbid learning disabilities.

### The Neuropsychology of Dyslexia

Approximately 10% of North American children experience developmental dyslexia (22, 23), a disorder characterized by difficulties with reading fluency that are not better explained by visual or cognitive impairments, psychosocial challenges, or poor language instruction. Observable symptoms include inaccurate or effortful reading, poor spelling ability, and the avoidance of leisure or work-related activities that involve reading (22, 24).

#### The Cognitive Profile of Reading Disability and Dyslexia

There are several overlapping theories about dysfunctional cognitive processes that impair reading fluency in developmental dyslexia (25), however three theories have garnered widespread support in current research literature: the *phonological deficit theory, double-deficit theory* and the *visual deficit theory*.

The *phonological deficit* theory is the most widely-promoted and well-established theory in dyslexia research (26, 27). This theory proposes that the core impairment in dyslexia is a deficit in phonological processing—a pervasive difficulty with forming associations between phoneme combinations and the correct corresponding sounds, known as grapheme–phoneme correspondence (28, 29). Deficits in phonological processing can be identified early in development (30, 31) and persist into adulthood (32, 33). Dyslexic children exhibit marked difficulties in manipulating *pseudowords* (non-sensical words made up of valid phonemes in a particular language), and display poor reading fluency when asked to read written words, but not when the words are read to them by another individual (6, 23). In addition, specific training to improve phonological processing leads to significant improvements in reading ability (34, 35).

The *double-deficit theory of dyslexia* builds on the notions presented in the phonological deficit theory. In addition to impaired phonological processing, this theory suggests dyslexia is characterized by a deficit in rapid automatized naming (RAN). RAN is the measure of how quickly an individual can recognize and name aloud a series of familiar objects, pictures, colors, or symbols, or letters (36). While recent studies have suggested that poor RAN performance may reflect impaired functional connectivity between brain structures that control visual processing and speech, poor RAN performance in sight-word reading can indicate phonological deficits in individuals with dyslexia and are more likely to underlie the difficulties in recognizing words (37, 38).

The *visual deficit theory* states that reading disabilities arise due to atypical development of the visual system, whereby there is disruption in the processing of visual information from letters and words in written text. Some neuropsychological studies have shown that individuals with reading disabilities exhibit impaired temporal processing, atypical eye movement regulation, and more frequent visual scanning errors in comparison to normal readers (39). While below-average performance on visual attention tasks in preschool has been shown to predict reading disability (40), it is unclear whether a visual system deficit is a root cause or a result of long-term reading disabilities (41), and seems to contradict recent findings of heightened visuospatial reasoning in dyslexic adults (42–44).

#### The Neuroanatomical Correlates of Dyslexia

**Figure 1:**
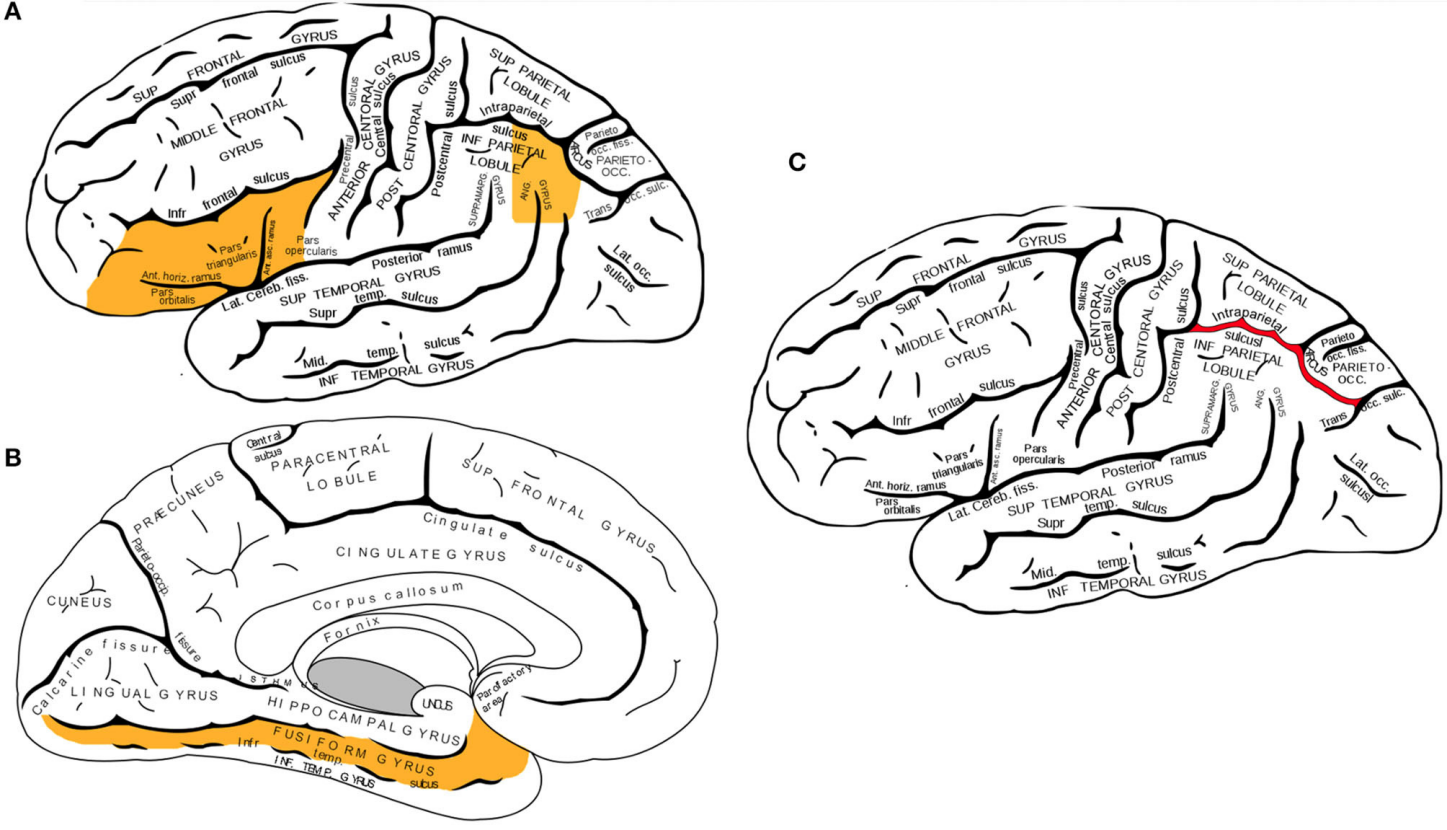
**(A,B)** Three neuroanatomical structures of the left hemisphere language processing network. **(A)** The left inferior frontal gyrus and the left angular gyrus are highlighted in this lateral view of the cerebral cortex. **(B)** Sagittal view of the cerebral cortex shows the left fusiform gyrus. **(C)** Lateral view of the left intraparietal sulcus. While activation of several brain regions correlates with various aspects of mathematical cognition, intraparietal sulcus (IPS) is the primary activation site during tasks that test numerical magnitude processing (see text). Brain diagrams were adapted from (45). Anatomy of the Human Body. Retrieved from https://www.bartleby.com/107/189.html.

Converging evidence from functional neuroimaging studies has pinpointed three neuroanatomical regions in the left hemisphere which primarily facilitate the multimodal processing of written words: the *left inferior frontal gyrus*, the *fusiform gyrus*, and *temporoparietal parietal junction* (displayed in Figures 1A,B). The inferior frontal gyrus contains *Broca's area*, a region that is well-known in neuropsychological literature for its mediating role in speech production, but less recognized for its role in processing phoneme sequences and phonological segmentation (46–48). The *fusiform gyrus* (also known as the *occipitotemporal gyrus*) contains the *Visual Word Form area*, which enables humans to distinguish between the symbols that form letters and numbers, and symbols that are otherwise arbitrary shapes (49, 50). The *temporoparietal junction* (a group of structures including the *angular gyrus, supramarginal gyrus*, and the *superior temporal gyrus*) facilitates semantic processing and is also involved in the analysis of phoneme sequences (16, 51, 52).

Dyslexia is associated with atypical development of the left hemisphere language network. Compared to age-matched controls, individuals with dyslexia show atypical physiological activity and white-matter connectivity in several frontal, parietal, and temporal structures in their dominant hemisphere (23, 26, 53). Studies that used functional neuroimaging to examine the neural correlates of phonological decoding consistently found that individuals with dyslexia typically exhibit lower cerebral blood oxygenation at the posterior regions of their language network—usually the left *fusiform gyrus* and the structures of the left *temporoparietal junction* (6, 16). Meta-analyses of neuroimaging studies that compared functional brain abnormalities between individuals with dyslexia have identified a variety of other brain regions that exhibit atypical activity during reading tasks. A meta-analysis of 28 studies identified hypoactivation of the left inferior frontal gyrus, left fusiform gyrus, left temporoparietal cortex, left occipitotemporal cortex, left precuneus, left frontal operculum, left precentral gyrus, and right superior temporal gyrus, as well as hyperactivation in the left anterior insula (54). Two other meta-analyses identified atypical hypoactivity in bilateral *superior temporal gyri*, left *middle* and left *inferior temporal gyri*, left *precuneus*, left *thalamus, right postcentral gyrus*, and the *right fusiform gyrus* during reading tasks (55, 56).

### The Neuropsychology of Dyscalculia

Developmental dyscalculia is characterized by difficulties in processing numerical information and performing basic mathematical operations, impeding the acquisition of age-appropriate mathematical skills (24, 57). It is estimated that dyscalculia affects 3–6% of the world population (58, 59), but dyscalculia is considerably unrepresented in research literature on learning disabilities [5, (57)]. Anywhere from 17 to 66% of individuals with dyscalculia also fit the diagnostic criteria for dyslexia (60, 61). Indeed, students with a math disability are just over two times more likely to also have a reading disability than those without a math disability (62).

#### The Cognitive Profile of Mathematical Disability and Dyscalculia

Dyscalculia is characterized by impaired *non-symbolic and symbolic numerical processing*, the ability to quickly estimate and manipulate numerical magnitudes and quickly perform mental operations without writing out procedures (63) or relying on verbally-based strategies such as counting (64, 65). The most popular view of mathematical cognition, and consequently of math disabilities [i.e., Triple Code Model; (66, 67)], entail that all development of symbolic number skills derive (through alternative format re-coding), and are ultimately grounded on the innate endowed ability of “number sense.” Accordingly, humans would form mental representations of numerical quantities using a *mental number line*, an imaginary line of numbers ordered in an ascending series. Thus, an individual would estimate the place any number or quantity on the number line and perform operations using their approximation of the number—a cognitive function known as *numerical magnitude processing* or the *approximate number system (ANS)*. The acuity of a person's ANS is often measured using *numerical magnitude comparison* tasks with *non-symbolic* quantities (e.g., a group of dots) as opposed to symbolic Arabic digits (e.g., the number 9). In a non-symbolic numerical comparison task, the individual is asked to approximate the correct place for non-symbolic quantity (without counting each item one-by-one) on a visually presented number line. A greater degree of error in ANS has been identified as the core deficit underlying developmental dyscalculia (68, 69). In comparison to typically-developing controls, children with dyscalculia demonstrate lower accuracy in approximating the number of non-symbolic items in a group, and lower accuracy in determining which group of items is greater in magnitude (70, 71). ANS is assumed to rely heavily on spatial representations of numbers (72, 73); individuals with dyscalculia often perform poorly on neuropsychological tests of visuospatial ability (74).

**Figure 2:**
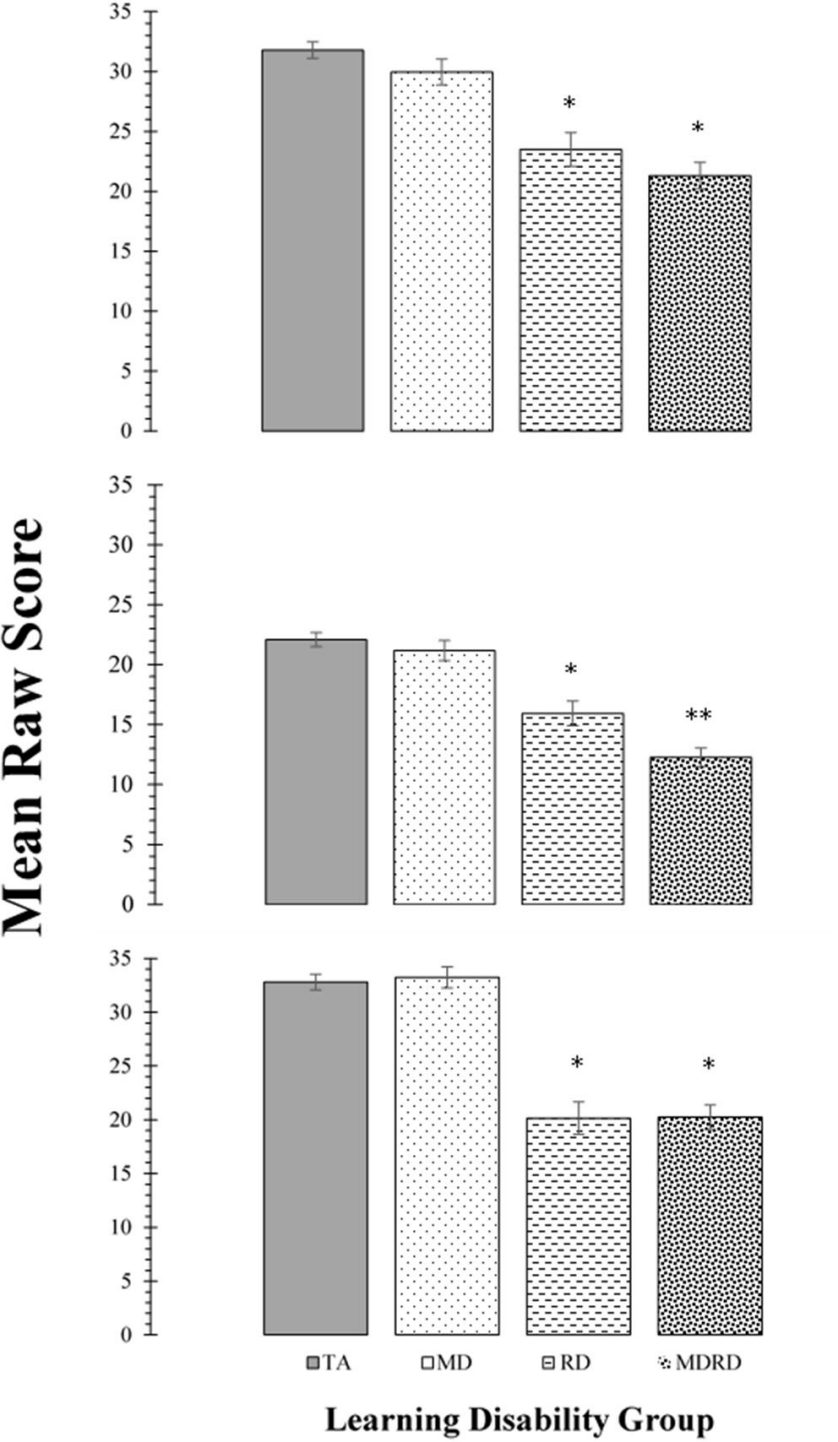
Top Panel: Mean scores for the Rosner Auditory Analysis task. Middle Panel: Mean scores for the Pseudowords Phoneme Deletion task Bottom Panel: Mean scores for the WRMT-R Word Attack subtest. For all panels, bars represent one standard errors. Asterisks summarize the results of *post hoc* Tukey test comparisons: “*” indicates a significant difference from the typical achievement (TA) control group at *p* < 0.05; “**” indicates a significant difference from the reading disability (RD) group at *p* < 0.05. TA > RD (Rosner Auditory Analysis: 8.29 [CI: 4.31 to 12.27]; Pseudowords: 6.15 [CI: 3.02 to 9.29]; Word Attack: 12.65 [CI: 8.53 to 16.76]; all *p*'s < 0.01). TA > MDRD (Rosner Auditory Analysis: 7.30 [CI: 7.30 to 13.64]; Pseudowords: 9.84 [CI: 7.34 to 12.43]; Word Attack: 12.56 [CI: 9.27 to 15.84]; all *p*'s < 0.01). For all other comparisons, RD = MD = MDRD.

However, a survey of the spectrum of quantity cognition for animals and humans (75) shows that number sense can directly account only for a fraction of the acquired skills, mainly involving approximate small (subitizing for numerosities 1–4) and large quantity assessment and comparison. Oral and written language account for most of the learning spectrum [see Figure 2 in (75)]. On this background, the “primacy” of the number sense has been most recently challenged, since the bulk of multidisciplinary existing evidence demonstrates that the alleged mapping between number sense and symbolic, more complex notions are not as direct as postulated (for example in the most influential Triple Code Model). A review of current neuroscience and behavioral evidence (76) suggests that several alternative possible and plausible routes of normal and atypical non-symbolic to symbolic correlations could occur which provide a better empirical account of math achievement than direct effects of number sense.

One perspective alternative to ANS contends that numerical ability is grounded on representing, understanding, and manipulating *symbol-symbol associations* (SSA). That is, small numerical symbols are initially mapped on a precise representation (e.g., the subitizing range) which, supported by increasing counting and linguistic competency, eventually leads to an independent and exact symbolic system based on order relations between symbols (77). Most magnitude estimation and comparison effects found in studies confirming ANS can be equally explained and modeled in terms of the SSA, and there is also sufficient evidence of distinct brain mechanisms associated to symbolic and non-symbolic numerical processing (78). Further, recent meta-analyses show that symbolic numerical processing tasks are a strong predictor of arithmetic and have consistently been found to be deficient in dyscalculia [see, for instance (79)]. Critically, representatives of this alternative view of numerical cognition differentiate between a non-symbolic deficit and an *access deficit* in dyscalculia, which reflects intact ANS, but deficient access to number semantics from numerical symbols (65).

At the same time, a wealth of evidence in research and practice shows that during formal schooling children with dyscalculia experience learning challenges in *symbolic and linguistic-based quantitative reasoning* related to academic mathematics such as arithmetic, not only numerical skills. These difficulties include learning and remembering exact number words and concepts, and applying skills in: addition/subtraction, multiplication and fraction strategies, commutation and percentages, using place value, as well as geometry, time, measurement, and word problems (80). Without discounting the important role that basic numerical processing might play, the scope of the present work is more narrowly focused on the latter higher-level symbolic and linguistically-based quantitative abilities as they are more directly linked with achievement in educational settings (64).

#### The Neuroanatomical Correlates of Dyscalculia

The current literature of brain imaging studies reveals that the brain recruits a wide variety of interconnected regions during mathematical tasks, including prefrontal, posterior parietal, occipito-temporal, and hippocampal areas (81). Neuroimaging studies on individuals with dyscalculia have identified two parietal regions associated with the manipulation of numerical quantities: the bilateral *intraparietal sulci* and the left *angular gyrus*. Multiple functional neuroimaging studies have shown that the right and left *intraparietal sulci* (shown in Figure 1C) become activated during calculation tasks that involve numerical magnitude processing (69, 82, 83). In contrast, the *angular gyrus* becomes activated during the retrieval of arithmetic facts from long-term memory, such as when finding the solutions to simple multiplications (84, 85).

While the neuroanatomical evidence of atypical brain function in developmental dyscalculia is not quite exhaustive, several functional neuroimaging studies have reported atypical activation patterns at the intraparietal sulci. Compared to age-matched controls, children with dyscalculia exhibit reduced activation at the right intraparietal sulcus when performing non-symbolic numerical comparison tasks [for example (86)]. In addition, applying TMS to the right intraparietal sulcus can severely impede performance on numerical magnitude tasks, artificially producing deficits that are equivalent to those observed in adults with dyscalculia [for example (87, 88)].

### An Overview of Cognitive Profiles of Comorbid Math and Reading Disabilities

A body of work has focused on the cognitive profiles of individuals with comorbid math and reading disabilities, establishing a design (here dubbed as the “four-groups design”) which has become pivotal in this research area. Specifically, Willburger et al. (89) examined cognitive performance in children with reading disability alone (RD), mathematical disability alone (MD), or with comorbidity of both disabilities (MDRD) as compared to typically developing and/or achieving children (TD and/or TA). MDRD children exhibited additive deficits in rapid automatized naming; this suggested that the deficits associated with comorbidity are additive and not qualitatively different from the deficits in the single disabilities. Later, this team (90) examined how domain-specific processes (e.g., symbolic and non-symbolic numerical processing, phonological processing) and domain-general processes (e.g., working memory, computations) contribute to comorbidity. MDRD children exhibited domain-specific deficits in phonological processing and numerical magnitude processing, performing at the same level as individuals with RD or MD. Unexpectedly, both the latter groups demonstrated better short-term working memory than the MDRD and the TD group, hinting that some domain-general processes may contribute to comorbidity.

Furthermore, Moll et al. (91) showed how three domain-general processes—namely processing speed, temporal processing, and verbal memory—can correlate differentially with reading and mathematical performance and are also associated with inattentive behavior. Both RD and MD children exhibited deficits in verbal memory. However, after controlling for parent-reported difficulties with inattention, deficits in verbal processing became associated with reading ability alone, whereas slowed temporal processing and visuospatial memory deficits were associated with mathematical ability alone. The authors concluded that deficits in processing speed, temporal processing, and verbal memory reflect variations in subclinical attention difficulties, and that reading and mathematical disabilities may thus be the outcome of multiple impaired cognitive systems rather than individual domain-specific processes. Relatedly, Wilson et al. (74) demonstrated that MDRD adults exhibited additive deficits in rapid naming and working memory, equivalent to the sum of the deficits exhibited by adults with the single disabilities. These authors concluded that additive domain-general deficits were likely correlates (not the underlying cause or the eventual consequence) of comorbidity.

More recent studies have shown that some processes traditionally considered as domain-specific may play an important role in comorbidity. Slot et al. (92) showed that children's rapid automatized naming and phonological awareness were associated with reading performance, whereas number sense and visuospatial working memory were associated with mathematical performance. However, phonological awareness was also predictive of mathematical performance, suggesting that a shared deficit in phonological processing may underlie both RD and MD. Similarly, Raddatz et al. (93) found that MD children showed deficits in various non-verbal and verbal tasks related to number processing, whereas RD children showed deficits in verbal tasks.

In contrast to this literature, neuroscience studies have rarely adopted the four-groups design. Improving on this limitation, the following study was designed to start filling some gaps in clarifying the nature of domain-specific and domain-general deficits of reading-math comorbidity with reference to the currently known neural underpinnings of dyslexia and dyscalculia.

### The Present Study: Research Questions, Design and Hypotheses

The primary objective of this study was to outline a neuroeducational model of dyslexia-dyscalculia comorbidity—a framework for understanding the psychoeducational and neuropsychological characteristics of individuals with comorbid math and reading disabilities that can be tested for validity in future research. The neuroeducational model proposed here is [as defined by (94)] a preliminary theory or set of hypotheses to synthesize current knowledge and then guide and refine evidence-based practice in education, public health and the allied fields. It should not by any means be interpreted as proof of established knowledge or theory. We fully expect this “probing” model to be tested and re-tested and in this process modified, refined, or even falsified based on future research. This model was established in three phases. In the first phase—using a psychoeducational approach—we examined the performance of individuals with math and reading disabilities on a series of psychoeducational tests and drew conclusions about the specific cognitive deficits they exhibited. In the second phase—using a neuropsychological approach—we performed a systematic review of existing clinical studies on the neuroanatomical correlates of dyslexia and dyscalculia; then, we identified the involvement of key neuroanatomical structures displaying abnormal function. In the third phase, we mapped the deficits as measured by psychoeducational tests to their most plausible neuroanatomical correlates obtained in the systematic review, creating a neuroeducational model of comorbidity that unites the broad psychoeducational definitions of math and reading disabilities with neuropsychological evidence of the biological characteristics of dyslexia and dyscalculia. This series of operations allowed us to build correspondence between the diagnostic tools used to identify learning disabilities in psychoeducational context and the neurodevelopmental theories of dyslexia and dyscalculia in the neuropsychological literature.

To determine the cognitive profile of comorbid dyslexia-dyscalculia, performances were measured from a sample population with math disability, reading disability, and dual math, and reading disability via a comprehensive battery of psychoeducational tests. The statistical analyses of their psychoeducational outcomes were used to (i) determine if there were any measurable cognitive deficits that were unique (in nature or in magnitude) to the participants with comorbid reading-math comorbidity; (ii) determine if the cognitive deficits were domain-specific (within the realm of reading or numerical cognition) or domain-general (working memory and/or executive functions outside the realm of reading or numerical cognition) in nature; (iii) determine the nature of the relationship between math and reading deficits in the comorbid group. Two sets of hypotheses were assessed:

*First set of hypotheses*: It was hypothesized that the deficits in the comorbid participants would either be *additive* (where the approximate sum of the deficits in the reading-disabled participants and the math-disabled participants is measured), *synergistic* (an over-additivity caused by an interaction between math and reading deficits), or *antagonistic* (an under-additivity caused by an interaction between math and reading deficits). The nature of the relationship between math and reading disabilities was determined using the same 2 × 2 factorial design previously used in the four-groups design literature (74, 90). A significant interaction between the math disability and reading disability indicates a synergistic over-additivity or an antagonistic under-additivity in the mean scores of a particular test, and lack thereof indicates an additive effect.

*Second set of hypotheses*: it was hypothesized that the comorbid participants in this study would exhibit: (1) impaired reading fluency and phonological processing equivalent to those shown by individuals with reading disability alone, (2) impaired quantitative reasoning skills equivalent to those shown by participants with mathematical disability alone, and (3) deficits in working memory equivalent to those shown by individuals with reading disability alone. Consequently, it was also hypothesized that, consistent with the proposed neuroeducational approach, it should be possible to derive a mapping of correspondence between the pattern found in the psychoeducational findings and known neuroanatomical correlates in the clinical neuroimaging literature, which can be empirically tested with further neuroimaging studies.

## Materials and Methods

### Psychoeducational Tests

#### Tests of Achievement for Identifying Math and Reading Disabilities

##### Wide Range Achievement Test 3rd edition (WRAT3) arithmetic subscale

The WRAT3 Arithmetic subscale is a test of written arithmetic problems, which included number addition, subtraction, multiplication, and problems involving fractions and decimals.

##### Wide Range Achievement Test 3rd edition (WRAT3) reading subscale

The *Reading subscale* is a single word reading test, where participants were asked to read aloud a series of increasingly difficult words.

#### Testing Phonological Processing

##### Rosner Auditory Analysis Test

The Rosner Auditory Analysis test is the first of two Phoneme Deletion tasks included in this study. Participants were instructed to repeat a list of 40 common English words. Next, the test administrator asked the participant to repeat each word without pronouncing a specific phoneme, thereby “deleting” the first, last or embedded phoneme from the word and pronouncing the word fragment(s) that remained.

##### Pseudowords Phoneme Deletion task

In this second Phoneme Deletion task, participants were instructed to listen to 30 pseudowords and then repeat it by “deleting” a specific phoneme.

##### Woodcock Reading Mastery Tests-Revised (WRMT-R) Word Attack subtest

The Word Attack subtest examines a participant's phoneme-grapheme awareness without relying on a verbal demonstration by the test administrator (95). Participants were instructed to read a list of 45 pseudowords. The level of difficulty gradually increased throughout the test; the number of syllables in each pseudoword increased intermittently from 1 syllable to 4 or 5 syllables by the end of the list.

#### Testing Quantitative Reasoning

##### KeyMath revised, interpreting data subtest

Participants completed the Interpreting Data subtest of the revised KeyMath Assessment (KeyMath-R) (96). Participants were tasked with solving a written mathematical problem (i.e., “Kareem can read sixty pages in two and one–half hours. How many pages can he read in 1 hour?”).

#### Testing Intellectual Functioning

All participants, aged 17 and over, completed three subtests of the Wechsler Adult Intelligence Scale (WAIS-R) (97). Participants aged 6 to 16 completed three analogous subtests from the Wechsler Intelligence Scale for Children (WISC-III) (98).

##### WAIS-R/WISC-III vocabulary subtest

The Vocabulary subtest measures a person's semantic memory retrieval. Participants performing the WAIS-R were asked to orally define a series of 30 vocabulary words, gradually increasing in difficulty. Participants performing the WISC-III were asked to name pictures representing each word.

##### WAIS-R/WISC-III Block Design subtest

In the Block Design subtest, participants were asked to re-create a model or a picture of a design using up to nine red and white blocks within a time limit. This test was included as a measure of visuospatial reasoning.

##### WAIS-R/WISC-III Digit Span subtest

The Digit Span subtest examines verbal working memory. Participants were presented orally with a series of single-digit numbers. In the first half of the trials, they were required to orally repeat the presented numbers in the same order they heard (forward digit span); in the second half of the trials, they were to repeat the presented numbers in the reverse order (backward digit span).

### Procedure

Participants were tested individually for a 3 h session (including two 10 min breaks). Each testing session began with the administration of the Vocabulary, Block Design, and Digit Span subtests from the WAIS-R or the WISC for participants aged 6–16. Successively, after the first break, each participant completed the Reading, Spelling, and Arithmetic subscales of the WRAT3. After the second break, each participant completed a series of psychoeducational tests. All participants completed four tests of phonological processing: The Rosner Auditory Analysis task, the Pseudowords Phoneme Deletion task, followed by the Word Attack and Word Identification subtests of the Woodcock Reading Mastery Tests (WRMT-R), the latter being excluded from this analysis. Lastly, participants completed the KeyMath Interpreting Data subtest.

### Sampling

The participants in this study were selected from a database resulted from a 10 year prospective cohort research study at the University of British Columbia (UBC). A total of 585 participants ranging from 7 to 77 years of age were recruited from around the Greater Vancouver Area as well as the graduate and undergraduate student population at UBC. They were recruited through a publicly advertised free comprehensive psychoeducational assessment offered as compensation for their participation and in exchange for use and publication of the resulting anonymous group data and findings. This study was approved by the UBC institutional research ethics boards in accordance with the 1964 Declaration of Helsinki ethical standards and in strict adherence of the Tri-Council Policy Statement (https://ethics.gc.ca/eng/policy-politique_tcps2-eptc2_initiatives.html). Participants or their parents/guardians (for children < 12 years of age), signed a consent form; parental/guardian's consent was conditional on children's active assent. Participants or parents/guardians completed a brief questionnaire on demographic and socioeconomic information about themselves or their family.

The testing format varied over the decade of data collection, and over 50 different types of test scores were entered into database. The initial database was reduced so as to only include the participants who completed specific psychometric tests in the same specific format and which therefore provided information about the cognitive profiles of individuals with dyslexia and dyscalculia permitting to test the objectives of this study.

Inclusion criteria for the present study entailed: (1) completion of The WRAT3 Arithmetic subscale and WRAT3 Reading subscale, which served as the main diagnostic indicators; (2) completion of domain-specific tests that examine phonological processing (the reading domain) or quantitative reasoning (the mathematical domain), and domain-general tests that examine executive functions (such as working memory and spatial reasoning). In the mathematical domain, only one test was selected: the Interpreting Data subtest of the revised KeyMath Assessment; (3) completion of three neuropsychological tasks that test domain-general cognitive functions were selected: The Vocabulary, Block Design, and Digit Span subtests of the WAIS-R (for participants ages 17 and older) or the WISC-III (participants ages 16 and younger); and finally (4) Estimated IQ scores [calculated using the sum of the WAIS-R Vocabulary subtest and the WAIS-R Block Design subtest, as in (12)] had to be > 70, which we adopted as the clinical threshold for low IQ (99).

The final analysis included data from *360* participants. The average age of the participants (on the day of testing) in each group are presented in Table 1. A one-way ANOVA followed by Tukey *post-hoc* multiple comparisons revealed that the average age of the RD group was significantly lower than the average age of the TA group (*p* = 0.008), the MD group (*p* = 0.017) and the MDRD group (*p* = 0.007). A two-way MANCOVA was conducted to examine the effect of age on the scores from all seven psychoeducational tests, with math disability and reading disability as the independent variables and age as a covariate. Using the Bonferroni procedure to correct for multiple ANOVAs (with a significant threshold of *p* < 0.008), there were no significant interactions between age and math disability, nor between age and reading disability, on the mean scores for any of the psychoeducational tests (Wilks' Lambda = 0.012). Furthermore, a three-way MANOVA was conducted with math disability, reading disability, and age category as independent variables. The participants were divided into two age categories: below age 16 and above age 16. MD × Age Category interaction was not significant (Wilks' Lambda = 0.780) nor was the RD x Age Category interaction Wilks' Lambda = 0.349). As a result, the low average age of the dyslexic did not appear to have a significant effect on the psychoeducational test results as whole.

**Table 1:** Characteristics of the four groups.

	**TA**	**MD**	**RD**	**MDRD**	**All groups**
N	158	69	46	87	360
Mean age (years)	26.47 (14.66)	26.80 (12.69)	19.22 (10.96)	27.22 (12.99)	25.79 (13.65)
*n* Female	77	35	16	47	175
% Female	48.73%	50.72%	34.78%	54.02%	48.61%
WRAT3 Arithmetic	57.82 (19.38)	13.62 (7.34)	50.26 (17.05)	10.34 (7.66)	36.84 (26.81)
WRAT3 Reading Mean Score	63.42 (19.24)	53.00 (17.18)	12.09 (8.20)	10.14 (8.11)	41.99 (28.77)
Estimated IQ	109.61 (14.28)	97.75 (13.45)	100.80 (16.42)	88.74 (13.57)	101.17 (16.51)
Education Rating	3.23 (1.41)	3.10 (1.29)	2.43 (1.19)	3.15 (1.22)	3.08 (1.31)
Occupation Rating	3.65 (1.30)	3.69 (1.53)	3.67 (0.87)	3.20 (1.44)	3.54 (1.37)
Median Income^a^ Rating	3.52 (1.63)	3.41 (1.36)	3.50 (1.34)	3.49 (1.28)	3.49 (1.48)

*Numbers in parentheses represent one standard deviation from the mean. Underlined numbers are mean percentile scores that are within the 25th percentile on the WRAT3 Arithmetic or WRAT3 Reading subtest*.

a *The distribution of income relative to the period studied was relatively stable in Vancouver and comparable to other big cities (>1M) in Canada (i.e., Toronto, Montreal, Ottawa, Calgary). Although the distribution was positively skewed around the mean (60–70 K in CND$) relative to other cities, because we do not intend to generalize our results to the entire Canada, what is most relevant is that there were no differences in income distribution between our four groups*.

Other MANOVA and MANCOVA analysis using a similar approach as the one used to investigate age effects showed no significant sex differences.

### Diagnostic Criteria and Subgroups

Using previously established cut-off criteria (15, 100), each participant was assigned to one of four groups: the *math disability (MD)* group (participants who scored 25th percentile or lower on the WRAT3 Arithmetic subscale), the *reading disability (RD) group* (25th percentile or lower on the WRAT3 Arithmetic subscale), the *comorbid math and reading disability (MDRD)* group (25th percentile or lower on both WRAT3 subscales), and the *typical achievement (TA)* control group (higher than the 25th percentile on both WRAT3 subscales). The mean percentile scores for each group and mean age of the participants (on the day of testing) are reported in Table 1.

Three measures of socioeconomic status (SES)—education, occupation, and median income—were evaluated in the present study (reported in Table 1). To measure SES, we used the Kuppuswamy's socioeconomic ranking scale validated for urban communities (101). Each participant received a numerical rating between 1 and 7 for the highest level of education they had achieved by the day of testing (1 = elementary school certificate or currently enrolled, 2 = middle school certificate, 3 = secondary school diploma, 4 = some college/university or post-secondary diploma, 5 = college/university degree, 6 = graduate degree, 7 = professional degree). Each participant received an individual rating for their occupation status (1 = unemployed, 2 = unskilled worker, 3 = semi-skilled worker, 4 = skilled worker, 5 = clerical, shop-owner, farmer, 6 = semi-profession, 7 = profession). For the participants ages 16 and younger, the highest level of occupation status achieved by either one of their parents was used as a proxy for their own occupation rating. Median household income ratings were generated for each participant by the postal code of the home address that they provided on the day of testing (1 = $50,000 or less, 2 = $50,000–$60,000, 3 = $60,000–$70,000, 4 = $70,000–$80,000, 5 = $80,000–$90,000, 6 = $90,000 or more), based on most temporally proximal Canadian population census data (102). Preliminary one-way ANOVAs and Tukey *post-hoc* multiple comparisons were conducted to identify any between-group differences in the three SES measures. There were no significant between-group differences in occupation or median income rating. There was only a significant difference in mean education rating *between* the TA and RD groups (*p* = 0.007); as previously noted, this is explained by the lower average age of the RD group, when the contrast on mean education rating was run controlling for age this difference was no longer significant.

### Statistical Analysis

The analysis involved two separate sets of tests for the first and second set of hypotheses (see section The Present Study: Research Questions, Design and Hypotheses). Relative to the first set of hypotheses, a two-way ANOVA was conducted for each of the seven psychoeducational tests, to assess just the *interaction* between math disability and reading disability across the four groups. The model followed a 2 × 2 factorial design, where the two between-subject factors were math disability (with two levels, math disability vs. no math disability) and reading disability (also two levels, reading disability vs. no reading disability). We followed the same procedures consolidated in previous studies on individuals with comorbid math and reading disabilities (74, 90) whereby, the interaction term serves as an indicator of the type of relationship between deficits in individuals with MDRD. A significant 2-way interaction between math disability and reading disability would indicate a synergistic or antagonistic relationship between math and reading disability—an over-additivity or under-additivity of deficits in domain-specific or domain-general cognitive processes. Main effects were irrelevant to the objectives of the study and are not considered, to avoid redundancy. Nonetheless, for rigor, they were counted in the correction for Type I error inflation due to multiple testing (see below). To assess the second set of hypotheses, one-way ANOVAs and *post-hoc* Tukey pairwise comparisons were used to analyze focused between-group differences in performance between TA, RD, MD, and MDRD groups, with each psychoeducational test measurement as dependent variable, and learning disability groups as levels of the independent variable/factor. This followed directly from the second set of hypotheses for this study.

To counteract Type I error inflation, we adopted the standard Bonferroni criterion; effects were deemed significant if p was below 0.00192; this corresponded to the *p*-value adjustment: 0.05/26 tests, which included all interaction and main effect tests of the two-way ANOVA as well as all one-way ANOVAs. Correspondingly, for the Tukey procedure, the same correction was applied to keep *p*-values below adjusted 0.05 level.

### Systematic Review of the Neuroanatomical Correlates of Dyslexia-Dyscalculia Comorbidity

The protocol for this review followed the guidelines established by the *Preferred Reporting Items for systematic Review and Meta-Analysis Protocols* (103); a detailed checklist with inclusion/exclusion criteria, search terms, and methods is presented in Table 2.

**Table 2:** PRISMA-P Protocol for Systematic Review (103).

Rationale	To identify any neuroanatomical structures whose atypical function may be associated with the cognitive deficits exhibited by individuals with dyslexia and dyscalculia
Objectives	The review answered the following questions: • “What brain regions show atypical activity in dyslexia alone?” • “What brain regions are show atypical activity in dyscalculia alone?” • “What brain regions are atypical activity in comorbid dyslexia-dyscalculia?
Eligibility criteria	Studies published in academic research journals since January 1, 2004. This marks the beginning of the current definition of specific learning disability (104) •The studies involved 20+ participants, males and females ages 6 and older •The studies followed a quasi-experimental design with at least two groups: one group with a learning disability (dyslexia or dyscalculia) and a control group •The studies did not involve individuals with any medical condition (other than dyslexia or dyscalculia) or any other life circumstance that could have influenced their performance on the cognitive tasks (ADHD, neurodegenerative disease, lack of education, etc.) •The investigators applied one of the three following techniques: a) Functional magnetic resonance imaging (fMRI) to examine physiological correlates of cognitive activity during phonological or numerical magnitude comparison tasks b) Diffusion tensor imaging (DTI) to examine structural differences in white or gray matter composition between key neurological structures c) Lesion-symptom mapping (caused by either a stroke or a brain tumor)
Information sources	•Google Scholar •American Psychological Association (PsycINFO) •Education Resources Information Center (ERIC) •NIH MEDLINE Database (PubMed) •Web of Science
Search strategy	Step 1: Preliminary Search A preliminary search was performed using Google Scholar to find the leading authors in learning disabilities research, identify their seminal publications on dyslexia, dyscalculia, and provide a working definition for each disorder Step 2: Existing Meta-Analyses A secondary search was performed to using PsycINFO and ERIC to identify studies that examined comorbid dyslexia-dyscalculia, and to identify any existing meta-analyses on the cognitive or neurological correlates of each learning disability Step 3: Detailed Search A detailed search of medical literature was performed using PubMed and Web of Science to identify empirical studies that used functional or structural MRI to examine individuals with (i) dyscalculia and (ii) dyslexia, and that report the neuroanatomical structures where atypical function, white matter composition, or functional connectivity is associated with each disorder a) A combination of the following search terms were used to identify functional and structural neuroimaging studies on dyslexia: “neurobiological dyslexia” “neurobiological reading disability” “brain region dyslexia” “brain region reading disability” “neuroimaging dyslexia” “neuroimaging reading disability” “fMRI dyslexia” “fMRI reading disability” “MRI dyslexia” “MRI reading disability” “DTI dyslexia” “DTI reading disability” b) A combination of the following search terms were used to identify functional and structural neuroimaging studies on dyscalculia: “neurobiological dyscalculia” “neurobiological math disability” “brain region dyscalculia” “brain region math disability” “neuroimaging dyscalculia” “neuroimaging math disability” “fMRI dyscalculia” “fMRI math disability” “MRI dyscalculia” “MRI math disability” “DTI dyscalculia” “DTI math disability” Step 4: Lesion-symptom Mapping Studies A second detailed search of medical literature was performed using PubMed and Web of Science to identify empirical studies that examined patients with alexia (acquired dyslexia) or acalculia (acquired dyscalculia), who had suffered damage to structures that mediate in math or reading ability, caused by an ischemic stroke or brain tumor a) A combination of the following search terms were used to identify lesion-symptom case studies of patients with alexia: “neurobiology AND acquired dyslexia” “neurobiology AND alexia” “lesion AND acquired dyslexia” “lesion AND alexia” “stroke AND acquired dyslexia” “stroke AND alexia” “brain tumor AND acquired dyslexia” “brain tumor AND alexia” b) A combination of the following search terms for acquired dyscalculia: “neurobiology AND acquired dyscalculia” “neurobiology AND acalculia” “lesion AND acquired dyscalculia” “lesion AND acalculia” “stroke AND acquired dyscalculia” “stroke AND acalculia” “brain tumor AND acquired dyscalculia” “brain tumor AND acalculia”
Study records	One independent reviewer selected the published studies that fit the eligibility criteria. The selected publications were legally stored and classified using Mendeley Desktop (Version 1.15.2) for Windows 10
Outcomes and prioritization	The desired outcome was a list of brain regions that are involved in dyslexia and dyscalculia. Priority was given to studies that included participants all four groups (participants with dyslexia, dyscalculia, comorbid dyslexia-dyscalculia, and controls)
Synthesis	The results of the systematic review are synthesized using a table as displayed below. Each brain region identified in the review is classified by learning disability (whether the region is associated with dyslexia, dyscalculia), as well as by the type of atypical functionality displayed (whether the brain region is generally more active or inactive in individuals with the learning disability). Of primary interest are the neuroanatomical structures whose atypical function is common to dyslexia and dyscalculia; these structures are underlined in the table

The systematic review was performed in four stages. The first stage was a preliminary search to identify well-cited authors on math and reading disabilities and the avenues for future research using Google Scholar. The second stage of review provided working definitions of dyslexia and dyscalculia using PsycINFO and the Education Resources Information Center (ERIC). The third stage served to identify the neural correlates unique to dyslexia, the neural correlates unique to dyscalculia, and the neural correlates that are shared between the disorders. A detailed search of biomedical literature was performed to examine evidence from four types of studies: (i) functional neuroimaging studies, (ii) structural neuroimaging studies, (iii) functional connectivity studies, and (iv) lesion-symptom mapping studies. This stage of the review was conducted using literature available through the Web of Science and the National Center for Biotechnology Information (PubMed). The fourth and final stage of the review was initially conducted in April 2015 and identified 26 empirical studies met inclusion criteria. This stage of the review was repeated in April 2020 to add updated findings from the literature; 24 additional empirical studies were identified.

The functional neuroimaging studies included compared the brain physiology of people with and without dyslexia during reading tasks or compared the brain physiology of people with and without dyscalculia while they performed mathematical tasks. The structural neuroimaging compared the white and/or gray matter volume in specific neuroanatomic regions among people with dyslexia and/or among people with dyscalculia and normal controls. Similarly, the functional connectivity studies used the same types of comparisons applying MRI tractography in multiple neuroanatomic regions. To support the model with direct evidence, the searches also identified empirical clinical studies that examined patients with *alexia* (acquired dyslexia) or *acalculia* following traumatic brain injury.

## Results

### Tests of Phonological Processing

Mean scores on the tests of phonological processing (Rosner Auditory Analysis: RAA, Pseudowords: PW, Word Attack: WA) are shown in the panels of Figure 2. The results of the two-way ANOVA did not reveal a significant interaction between math disability and reading disability for any of these tasks (all *F*'s < 2.89; *p* > 0.10, $\eta_{\text{p}}^{2}$ ≤ 0.01). The one-way ANOVA identified a significant effect of learning disability group on the mean scores of the RAA [*F*_(3, 356)_ = 28.90, MSE = 2448.46, *p* < 0.0001, η_*p*_^2^ = 0.196], PW [*F*_(3, 356)_ = 39.28, *MSE* = 2067.86, *p* < 0.0001, η_*p*_^2^ = 0.25], and WA [*F*_(3, 356)_ = 49.92, *MSE* = 4528.74, *p* < 0.0001, η_*p*_^2^ = 0.30]. For all three tasks, Tukey *post-hoc* multiple pairwise comparisons revealed a similar pattern of significant intergroup mean scores differences, showing TA > RD, and TA > MDRD (all *p*'s < 0.01). For all other comparisons, RD = MD = MDRD (For more details see Note in Figure 2).

### The KeyMath Interpreting Data Subtest (Henceforth, *KeyMath-ID*)

**Figure 3:**
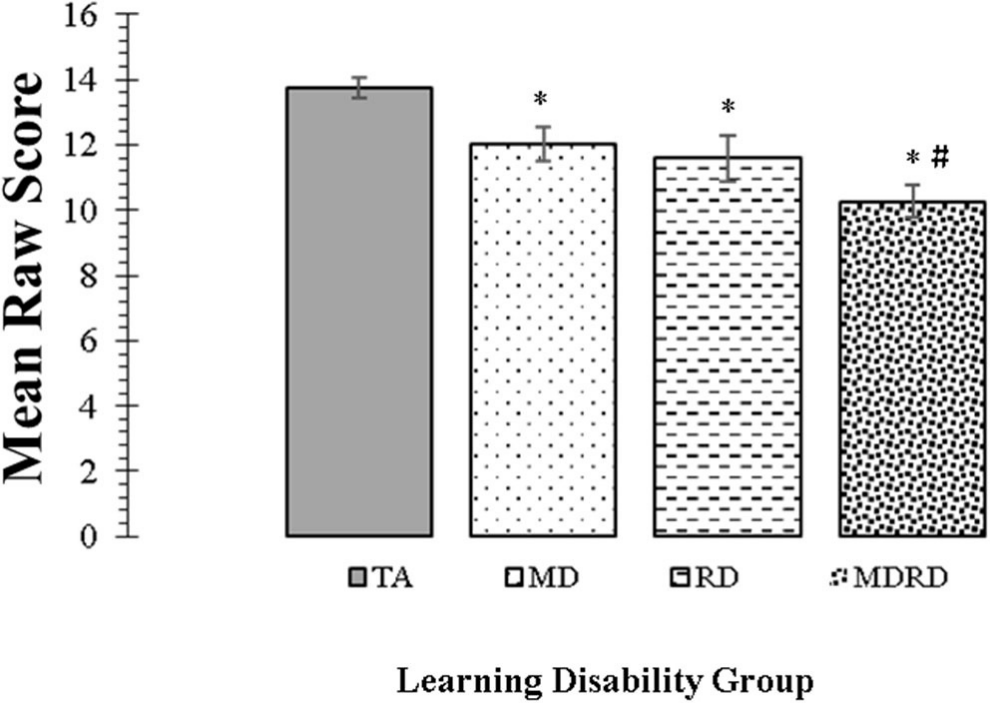
Mean scores for the KeyMath Interpreting Data subtest. Bars represent one standard errors. The symbol “*” indicates a significant difference from the typical achievement (TA) control group at *p* < 0.05 on *post hoc* Tukey test. The symbol “#” indicates a marginally significant trend at 0.05 <*p* < 0.10 on *post hoc* Tukey test. TA > MD (1.72, [CI: 0.07 to 3.36], *p* = 0.04). TA > RD (2.16, [CI: 0.25 to 4.07], *p* = 0.02). TA > MDRD (3.47, [CI: 1.95 to 4.99], *p* < 0.01). RD = MD. RD = MDRD. # MD > MDRD (1.75, [CI: −0.08 to 3.59], *p* = 0.068).

Mean scores and standard errors for the KeyMath-ID are shown in Figure 3. The two-way ANOVA did not reveal a significant interaction between math disability and reading disability (*F* < 1, *p* = 0.73, η_*p*_^2^ = 0.01). The one-way ANOVA identified a significant effect of learning disability group on the level of performance [*F*_(3, 356)_ = 12.23, *MSE* = 238.550, *p* = 0.0001, η_*p*_^2^ = 0.093]. Tukey comparisons revealed that the mean scores of the TA group were significantly higher than all other disability groups (TA> MD, TA > RD, and TA > MDRD (all *p*'s < 0.05). However, there were no other significant differences among the mean scores of the groups of individuals with disabilities, that is, RD = MD = MDRD (For more details see Note in Figure 3).

### Tests of Intellectual Functioning

**Figure 4:**
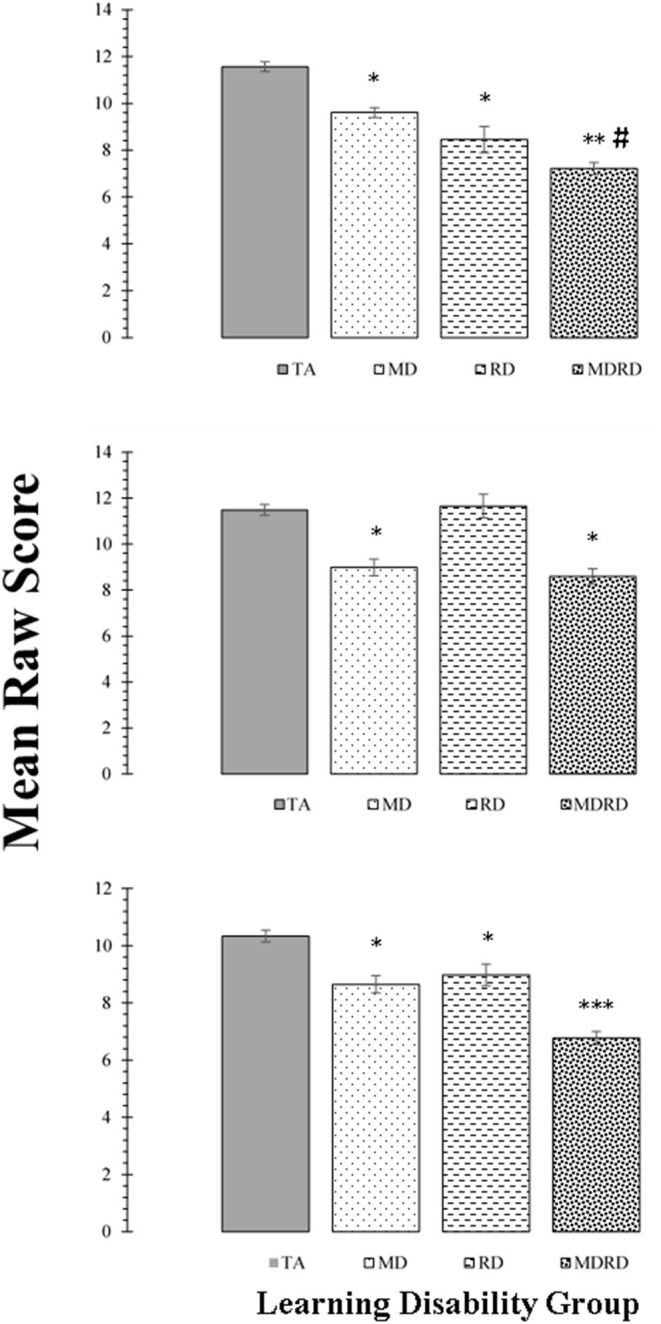
Top Panel: Mean scores for the WAIS-R Vocabulary subtest. Middle Panel: Mean scores for the WAIS-R Block Design subtest. Bottom Panel: Mean scores for the WAIS-R Digit Span subtest. For all panels, bars represent one standard errors. Asterisks summarize the results of *post hoc* Tukey test comparisons: “*” difference from the typical achievement (TA) control group at *p* < 0.05; “**” significant difference from the mathematical disability (MD) group at *p* < 0.05; “***” significant difference from the reading disability (RD) group at *p* < 0.05. The symbol “#” indicates a marginally significant trend at 0.05 < *p* < 0.10 on *post hoc* Tukey test. For the Vocabulary subtest: TA > MD (1.95, [CI: −0.92 to 2.98], *p* < 0.01); TA > RD (3.10, [CI: 1.90 to 4.30], *p* < 0.01); TA > MDRD (4.34, [CI: 3.39 to 5.29], *p* < 0.01). MD > MDRD (2.39, [CI: 2.39 to 3.54], *p* < 0.01). # RD > MDRD (1.24, [CI: −0.06 to 2.54], *p* = 0.069). For the Block Design subtest: TA > MD, (2.50, [CI: 1.36 to 3.64], *p* < 0.01); TA > MDRD (2.89, [CI: 1.84 to 3.94], *p* < 0.01). For the Digit Span subtest: TA > MD (1.68, [CI: 0.74 to 2.62], *p* < 0.01); TA > RD (1.36, [CI: 0.26 to 2.45], *p* = 0.01); TA > MDRD (3.57, [CI: 2.69 to 4.44], *p* < 0.01). MD > MDRD (1.88, [CI: 0.83 to 2.93], *p* < 0.01). RD > MDRD (2.21, [CI: 1.02 to 3.40], *p* < 0.01).

Mean scores on the tests of intellectual functioning (WAIS-R Vocabulary, Digit Span, and Block Design) are shown in the panels of Figure 4. The two-way ANOVA did not reveal a significant interaction on any of these subtests [*F*_(1, 356)_ < 1.33, *p* > 0.25, η_*p*_^2^ ≤ 0.02]. The one-way ANOVA identified a significant effect of learning disability group on the mean scores of the Vocabulary subtest [*F*_(3, 356)_ = 50.14, *MSE* = 383.92, *p* < 0.0001, η_*p*_^2^ = 0.30], the Block Design subtest [*F*_(3, 356)_ = 24.23, *MSE* = 225.95, *p* < 0.0001, η_*p*_^2^ = 0.17], and the Digit Span subtest [*F*_(3, 356)_ = 37.75, *MSE* = 240.86, *p* < 0.0001, η_*p*_^2^ = 0.24].

For the Vocabulary subtest, *post-hoc* tests showed that TA had higher mean scores than all the disability groups (TA > MD, TA > RD, and TA > MDRD (all *p*'s < 0.01). The mean difference between MD and MDRD was also significant (*p* < 0.01). For the Block Design subtest, only the mean differences TA > MD, and TA > MDRD were significant (*p* < 0.01).

On the Digit Span subtest, TA had higher mean scores than all the disability groups (TA > MD, TA > RD, and TA > MDRD, all *p*'s < 0.01). MDRD had significantly lower mean scores than MD and RD all *p*'s < 0.01) (For more details see Note in Figure 4).

### Systematic Review Report: Mapping Reading and Mathematical Disability Deficits to Neuroanatomical Correlates of Dyslexia and Dyscalculia

Table 3 lists all the studies recognized in the systematic review which identified neuroanatomical correlates of dyslexia and/or dyscalculia. It included studies published since 2004 which used either (a) functional neuroimaging to examine the cerebral blood oxygenation in individuals with dyslexia and dyscalculia, who performed during phonological or numerical magnitude tasks; (b) structural neuroimaging to examine white or gray matter tractography in individuals with dyslexia or dyscalculia; (c) lesion-symptom mapping in individuals who suddenly developed alexia (acquired dyslexia) or acalculia (acquired dyscalculia) after suffering a stroke or recovering from surgery to remove a brain tumor. The neuroanatomical correlates of dyslexia and the neuroanatomical correlates of dyscalculia are summarized in Table 4.

**Table 3:** Systematic review of the Neuroanatomical correlates of dyslexia and the neuroanatomic correlates of dyscalculia.

**Method**	**Neuroanatomical correlates of dyslexia**	**Neuroanatomical correlates of dyscalculia**
Functional Neuroimaging Studies (fMRI, PET)	**Hypoactivation during phonological tasks**: •Left superior frontal gyrus (105) •Left middle frontal gyrus (105) •***Left inferior frontal gyrus*** (106–108) •Left superior temporal gyrus (107, 109–111) •Left superior temporal sulcus (107, 109–112) •Left middle temporal gyrus (113) •Left inferior temporal gyrus (108, 109, 114) •***Left fusiform gyrus*** (112, 114–116) •Left superior parietal cortex (117) •Left inferior parietal cortex (117) •***Left angular gyrus*** (105, 109, 110, 118, 119) •Left supramarginal gyrus (120, 121) •Left middle occipital gyrus (112) **Hyperactivation during phonological tasks:** •Right medial prefrontal cortex (113) •Left primary motor cortex (109, 113) •Left anterior insula (109, 110, 113) •Left caudate nuclei (109, 110) •Lobule VI of the Left cerebellum (122) •Precuneus (112) No significant differences in activation between individuals with dyslexia and controls •Cerebellum (123) **Null Findings:** No significant differences in activation between individuals with dyslexia and controls during phonological tasks (129)	**Hypoactivation during numerical magnitude tasks**: •Left superior frontal gyrus (124) •Left medial prefrontal cortex (125) •Right fusiform gyrus (124, 125) •***Right intraparietal sulcus*** (125) •***Bilateral intraparietal sulci*** (124, 126, 127) **Hyperactivation during numerical magnitude tasks**: •Right superior frontal gyrus (124) •Left postcentral gyrus (124) •***Left angular gyrus*** (124, 126, 128) •Bilateral supramarginal gyrus (124) **Null findings**: No significant differences in activation between individuals with dyscalculia and controls during numerical magnitude tasks (129)
Structural Neuroimaging Studies (MRI)	**Lower gray matter volume vs. controls**: •Right middle frontal gyrus (130) •***Left inferior frontal gyrus*** (115, 131) •Left inferior temporal gyrus (115) •***Left fusiform gyrus*** (115, 132) •***Left angular gyrus*** (115, 132) •Occipitotemporal cortex (133) **Greater cortical thickness vs. controls**: •Right superior temporal gyrus (134) •***Left fusiform gyrus*** (134) No difference in gray matter volume between individuals with dyslexia and controls in the following regions •Cerebellum (135)	**Lower gray matter volume vs. controls**: •Left fusiform gyrus (132) •***Left angular gyrus*** (132) •***Right intraparietal sulcus*** (136) **Null findings**: No difference in matter volume between individuals with dyslexia and controls (135)
Functional Connectivity Studies (DTI)	**Lower functional connectivity in the following white matter tracts vs. controls**: •Right superior longitudinal fasciculus (137) •Left arcuate fasciculus, connecting ***the left inferior frontal gyrus*** and the left auditory cortex (138) •Reduced connectivity between the ***left inferior frontal gyrus*** and multiple left posterior temporal areas, including the ***left fusiform gyrus***, left inferior temporal gyrus, left middle temporal gyrus, and left superior temporal gyrus (139) •White matter tracts between the right parahippocampal gurus the ***left fusiform gyrus*** (140) •White matter tracts between the ***left angular gyrus*** and left lingual gyrus, as well as the ***left angular gyrus*** and the left cerebellum (105) •Left auditory thalamus and the left planum temporale (141) •***Left angular gyrus*** and left superior temporal gyrus (123) **Greater functional connectivity between the following structures among individuals with dyslexia vs. controls**: •Left cerebellum and the left supramarginal gyrus (122) •Thalamus and the inferior parietal cortex (117)	**Lower functional connectivity in the following white matter tracts vs. controls**: •Inferior fronto-occipital fasciculus and inferior longitudinal fasciculus, connecting the right fusiform gyrus and ***right intraparietal sulcus*** (145) •Bilateral posterior superior longitudinal fasciculus (146) **Greater functional connectivity between the following structures among individuals with dyscalculia vs. controls**: •***Bilateral intraparietal sulci*** and the left superior frontal gyrus (147) •***Bilateral intraparietal sulci***, the right superior temporal gyrus, and the right supramarginal gyrus (148) •Primary visual cortex and inferior occipital cortex (129) •Primary visual cortex and fusiform gyrus (129) **Null findings**: No significant differences between individuals with dyslexia and typical readers in the following white matter tracts: •Bilateral arcuate fasciculus (142) •Corona radiata (142)
	**Null findings**: No significant differences between individuals with dyslexia and controls in the following white matter tracts: •Bilateral arcuate fasciculus (142) •Corona radiata (142–144)	
Lesion-Symptom Mapping Studies	**Alexia associated with damage to**: •***Left inferior frontal gyrus*** (115, 149, 150) •Right Posterior middle temporal gyrus (151) •Right fusiform gyrus (152) •***Left fusiform gyrus*** (149, 153–155) •***Left angular gyrus***, via the posterior cerebral artery (156, 157) •Left supramarginal gyrus (158)	**Acquired dyscalculia associated with damage to**: •Left thalamus (159, 160) •***Left angular gyrus*** (161, 162) •***Left intraparietal sulcus*** (163)

*The **bolded and italicized** brain regions were identified as a neuroanatomical correlate of dyslexia or dyscalculia in all four types of studies in included in the systematic review (functional neuroimaging studies, structural neuroimaging studies, functional connectivity studies, and lesion-symptom mapping studies)*.

**Table 4:** Summary of the neuroanatomical correlates of dyslexia and the neuroanatomical correlates dyscalculia.

**Type of research study**	**Neuroanatomical correlates of dyslexia**	**Neuroanatomical correlates of dyscalculia**
Functional Neuroimaging Studies	Left angular gyrus Left inferior frontal gyrus Left fusiform gyrus	Left angular gyrus Left intraparietal sulcus Right intraparietal sulcus
Structural Neuroimaging Studies	Left angular gyrus Left inferior frontal gyrus Left fusiform gyrus	Left angular gyrus Right intraparietal sulcus
Functional Connectivity Studies	Left angular gyrus Left inferior frontal gyrus Left fusiform gyrus	Left angular gyrus Left intraparietal sulcus Right intraparietal sulcus
Lesion-Symptom Mapping Studies	Left angular gyrus Left inferior frontal gyrus Left fusiform gyrus	Left angular gyrus Left intraparietal sulcus

*The brain regions listed here have been characterized as neuroanatomical correlates of dyslexia or dyscalculia in all four types of studies included in this systematic review (functional neuroimaging studies, structural neuroimaging studies, functional connectivity studies, and lesion-symptom mapping studies), with the exception of the intraparietal sulci. The review did not identify any structural neuroimaging studies that characterized the left intraparietal sulcus as a neuroanatomical correlate of dyscalculia, nor did it identify any lesion-symptom mapping studies that characterized the right intraparietal sulcus as a neuroanatomical correlate of dyscalculia*.

#### Neuroanatomical Correlates of Reading Disability Deficits in Dyslexia

The systematic review identified a total of 22 different brain regions whose dysfunction or abnormal development has been associated with impaired reading among individuals with dyslexia and/or alexia. Of those 22 brain regions, only three were identified as neuroanatomical correlates of reading disorders in all four types of research studies surveyed. That is, multiple functional neuroimaging, structural neuroimaging, functional connectivity, and lesion-symptom mapping studies revealed that abnormal activity or development in the *left inferior frontal gyrus*, the *left fusiform gyrus* and the *left angular gyrus* likely contribute to impaired reading.

Individuals with dyslexia exhibit less activation than normal readers at the left inferior frontal gyrus (106–108), left superior temporal gyrus (107, 109–111, 164), left fusiform gyrus (114, 115), and the left angular gyrus (109, 110, 118, 119) when performing the same phonological tasks used to assess in individuals with reading disabilities.

Structural neuroimaging studies have also shown that individuals with dyslexia exhibit reduced gray matter in the left inferior frontal gyrus, fusiform gyrus, and angular gyrus (115). Boets et al. (138) determined that normal readers store phonological representations of words in the left auditory cortex (Brodmann Area 41 and 42), and that a functional connection between the auditory cortex and the left inferior frontal gyrus allows normal readers to access these representations to read more fluently.

Boets and colleagues also used diffusion tensor imaging to show that the left arcuate fasciculus—the bundle of axons that connect the inferior frontal gyrus to the auditory cortex—has significantly lower white matter in individuals with dyslexia than in controls, while the structure of the auditory cortex itself was left intact. The authors suggested that individuals with dyslexia develop below average ready fluency in part due to impaired access to phonological representations of words—even words that they are familiar with, exactly as in individuals with reading disabilities. More recent studies have shown the that individuals with dyslexia exhibit lower functional activation than normal readers in the white matter tracts between the left angular gyrus and left lingual gyrus (105) as well as the tracts between the left angular gyrus and left superior temporal gyrus (123).

Finally, lesions to the left fusiform gyrus can result in alexia (50, 149, 153, 154) or from damage to the left posterior cerebral artery, which supplies blood to the left angular gyrus (156, 157). Lesions to the *pars opercularis* section (Brodmann area 44) of the left inferior frontal gyrus can cause alexia, primarily in the form of abrupt deficits in decoding pseudowords (115, 149), whereas lesions to the *pars triangularis* section (Brodmann area 45) are well-known cause in Broca's aphasia (46).

#### Neuroanatomical Correlates of Mathematical Disability Deficits in Dyscalculia

The systematic review identified a total of 17 different brain regions whose dysfunction or abnormal development has been linked to impaired mathematical cognition among individuals with dyscalculia and/or acalculia. Of these 17 brain regions, converging evidence from multiple sources of evidence suggests that there are only two key neurological structures whose dysfunction may produce distinct and pervasive difficulties with mathematical cognition in individuals with dyscalculia: the *left angular gyrus* and the *bilateral intraparietal sulci*.

Atypical hypoactivation at the right intraparietal sulcus and atypical hyperactivation at the left angular gyrus have been associated with specific aspects of impaired mathematical cognition in individuals with dyscalculia. Individuals with dyscalculia exhibit less activation than controls at the bilateral intraparietal sulci (124–127) when performing variants of the non-symbolic magnitude comparison tasks used to assess the quantitative reasoning skills of individuals with mathematical disabilities. They exhibit greater activation than controls at the left angular gyrus when performing these same magnitude comparison tasks (124, 126).

Few structural neuroimaging studies have investigated the neuroanatomical correlates of dyscalculia. There is some evidence, however, that individuals with dyscalculia exhibit lower gray matter volume than controls in the right intraparietal sulcus (136) as well as in the left fusiform gyrus and left angular gyrus (132).

Individuals with dyscalculia show lower functional connectivity than controls between the right intraparietal sulcus and the right fusiform gyrus (145), and between the parahippocampal gyrus and left fusiform gyrus (140). More recent evidence suggests that individuals with dyscalculia show greater functional connectivity than controls between the bilateral intraparietal sulci and the left superior frontal gyrus (147, 148) as well as between the primary visual cortex and the fusiform gyrus (129).

Finally, lesions to the left intraparietal sulcus (163) or to the left angular gyrus (161, 162) can produce symptoms of acquired dyscalculia. Furthermore, direct damage to left angular gyrus from strokes typically results in *Gerstmann's syndrome*, a disorder characterized by a sudden inability to write (agraphia), an inability to recognize ones' own fingers (finger agnosia), left-right disorientation, and a severe impairment performing mathematical tasks (165).

In summary, the evidence concerning the role of the intraparietal sulci is mixed. The bilateral intraparietal sulci have been reported as correlates of impaired mathematical cognition in multiple functional neuroimaging and functional connectivity studies. However, the right intraparietal sulcus alone has been linked to dyscalculia in structural neuroimaging studies, whereas the left intraparietal sulcus alone has been linked to acalculia in lesion-symptom mapping studies. The left angular gyrus is the only neuroanatomical structure where abnormalities in physiological activation, white matter volume, white matter tractography, and lesions have been consistently identified in individuals with dyscalculia and/or acalculia. Furthermore, the left angular gyrus is the only neuroanatomical structure identified in this systematic review whose dysfunction is also linked to impaired reading in people with dyslexia or alexia.

## Discussion

### The Cognitive Profiles of Mathematical Disability, Reading Disability, and Comorbid Math and Reading Disability

Table 5 presents a summary of the cognitive profiles of MD, RD, and MDRD participants in this study, organized by their psychoeducational domain. In the reading domain, the RD and MDRD groups demonstrated impaired phonological processing. Similarly, in the math domain, the MD and MDRD groups demonstrated impaired quantitative reasoning on the KeyMath-ID subtest. However, unexpectedly, the RD group also demonstrated a deficit in quantitative reasoning, equal in magnitude to the two math-disabled groups. In the domain-general tests, the MD and RD groups demonstrated impaired verbal semantic memory and working memory; the MD group also demonstrated impaired visuospatial reasoning. Meanwhile, the MDRD group demonstrated additional severe impairments in verbal semantic memory and working memory.

**Table 5:** The cognitive profiles of the MD, RD, and MDRD participants.

**Domain**	**MD group**	**RD group**	**MDRD group**
Reading	N/A	•Impaired phonological processing	•Impaired phonological processing
Math	•Impaired quantitative reasoning	•Impaired quantitative reasoning^*^	•Impaired quantitative reasoning
Domain- general	•Impaired verbal semantic memory •Impaired verbal working memory •Impaired visuospatial reasoning	•Impaired verbal semantic memory •Impaired verbal working memory	•Impaired verbal semantic memory+ •Impaired verbal working memory+ •Impaired visuospatial reasoning

The symbol

* *indicates an unexpected finding. The symbol + indicates an additive deficit, where the MDRD group demonstrated a significantly greater deficit than either the MD or RD group*.

### The Additive Hypothesis of Cognitive Deficits in Comorbid Math and Reading Disabilities

The present analysis did not reveal an interaction between reading disability and math disability on any of the tests used. Therefore, the results do not support a synergistic or the antagonistic deficit hypothesis, echoing previous findings from children (90) and adults (74). Instead, these results lend further support to the additive hypothesis of cognitive deficits: comorbid learning disabilities are the sum result of separate, specific, underlying cognitive deficits. Importantly, although cross-sectional, the results are similar in children and adult groups. A remaining issue is to understand the mixed bag of domain-specific or domain-general effects.

### Domain-Specific Deficits in Comorbid Mathematical and Reading Disabilities

The contrast patterns determined in the univariate analysis only partially correspond with the domain-specific view of learning disabilities. On the three phonological tests, the RD and the MDRD participants both had lower performance scores than the TA participants, exhibiting a considerable and specific deficit in phonological processing. Meanwhile, the MD participants did not exhibit a phonological deficit. This result follows the well-established predictions from a long history of research in dyslexia; it has been shown for several decades that individuals with reading disabilities alone or comorbid reading-and-math disabilities consistently exhibit deficits in phonological processing, frequently making mistakes in applying phoneme-grapheme correspondence rules (28, 29, 31). It is this specific deficit in phonological processing that underlies poor word recognition, and thereby underlies poor reading fluency (6, 23). Consistent with the domain-specific hypothesis, both individuals with reading disabilities and those with the comorbid condition exhibited distinct impairments in phonological processing, but the impairment in individuals with comorbidity was no more severe than in individuals with single disabilities.

However, the results from the KeyMath-ID are not compatible with the domain-specific view of learning disabilities. Against the prediction of that view (70, 166), in our study individuals with reading disabilities showed significant deficits on tests of quantitative reasoning. The results do not support the additive hypothesis of cognitive deficits either, because the deficit demonstrated by the MDRD group did not equal the sum of the deficits in the MD and RD groups. A plausible account for the pattern of results is that successful completion of the KeyMath-ID relies on higher-level, general-domain skills such as verbal and linguistic reasoning (a conclusion supported by the fact that the KeyMath-ID correlates with tests such as the Kaufman Test of Educational Achievement, the Iowa Tests of Basic Skills, the Measures of Academic Progress test, and Group Math Assessment and Diagnostic Evaluation test).

It must be noted that the KeyMath-ID is not finely tuned enough to measure the very basic (“bottom-up”) mechanisms involved in numerical processing. Rather, it can validly identify shared linguistic, comprehension and reasoning deficits in comorbid mathematical and reading disabilities. We acknowledge the limitation in the completeness of our study, since performance on this subtest may not accurately reflect the role played by latent deficits in non-symbolic and symbolic numerical processing. However, despite these limitations, our findings clearly show all three learning disability groups demonstrated challenges in successfully solving the written word problems included in the KeyMath-ID. Importantly, a partial correlation analysis controlling for age and all demographic variables of our sample reveals a strong correlation with the WRAT-R arithmetic subtest (partial *r* = 0.78; *p* < 0.0001), indicating 60% of shared variance with the KeyMath-ID. In addition, cognitive research (167–169) has shown the WRAT arithmetic (which builds up from basic to progressively more complex numerical skills, i.e., from enumeration to pre-algebra) and more in general, arithmetic tasks, indeed tap onto symbolic quantitative skills, partially dependent on symbolic numerical processing [for critical discussion of these relationships, see (75, 78, 79)]. These converging elements therefore indicate, although indirectly, the plausible underlying constraint of number processing on the quantitative reasoning abilities we measured.

### Domain-General Deficits in Comorbid Reading and Mathematical Disability

The contrast patterns determined in the univariate analysis of the WAIS-R/WISC-III, Block Design, Vocabulary, Digit Span subtests correspond to a greater extent with the domain-general view of specific learning disabilities. On the Block Design subtest, the MD and MDRD groups both performed far below the typical achievement level, suggesting that individuals with mathematical disabilities experience a pervasive deficit in visuospatial reasoning. When performing a test of visuospatial reasoning such as the Block Design task, the participants make visual approximations about a group of shapes and how they can be rearranged to match a two-dimensional geometric pattern. Such approximations may be negatively influenced by impaired access to the approximate number system—the cognitive mechanism that allows to make estimations about objects and other non-symbolic quantities, and manipulate them in mathematical operations (63). This cognitive system is persistently impaired in individuals with mathematical disabilities (68, 69) and is correlated with impaired perceptual reasoning skills (72). Particularly, neuroimaging studies using the Corsi Blocks task (a test of visuospatial pattern recognition) reported a strong correlation between deficits in visuospatial ability and math disability (73, 170).

In contrast to the MD group, the RD group did not demonstrate a deficit in visuospatial reasoning; remarkably, they scored higher on average than participants in typical achievement range. Individuals with reading disabilities have previously shown to match the performance of non-learning-disability participants on various iterations of this visuospatial reasoning task (44, 90). It has been proposed that reading-disabled individuals may compensate for deficits in phonological processing by relying on visuospatial reasoning to learn, recognize, and articulate words (42, 43). However, while RD individuals have consistently shown faster response times (but not greater accuracy) in identifying impossible figures and manipulate complex shapes with blocks, there is insufficient evidence that they possess an advantage in spatial processing (171).

The most noteworthy findings of the current study were obtained from the WAIS-R Vocabulary and Digit Span subtests. On the Vocabulary subtest, the MD group and the RD group performed at a statistically equivalent level; both groups scored significantly lower on average than the typical achievement range, implying that both groups experienced a specific difficulty in articulating age-appropriate word definitions. This was an unexpected finding for the MD group in particular; individuals with math disabilities are not known to demonstrate difficulty in word recognition, especially when tested with familiar words. The difficulty demonstrated by the RD group was also unexpected, because the Vocabulary subtest does not directly assess phonological processing. The participants did not need to read any of the items (they were read by a test administrator), nor were any of the words considered to be too irregular for their age-appropriate lexicon (the WISC included common words such as “clock,” or “alphabet”). Given that the between-group differences in education rating were insignificant and none of the participants had an estimated IQ within the clinically-critical range, these findings were unexpected.

It was originally hypothesized that individuals with comorbid math and reading disabilities would not exhibit additive deficits on the Vocabulary subtest because it does not exclusively assess domain-specific phonological processing or arithmetic abilities. The MDRD participants performed at an even lower level than both the MD and the RD group. While the mean difference between the RD and MDRD was marginally significant (*p* = 0.069), the difference in mean scaled scores between the TA and MDRD groups (4.34) was nearly the sum of the difference between the TA and MD groups (1.95) and the difference between the TA and RD groups (3.10), corresponding to the additive hypothesis. In stark contrast, the individual deficits shown by the MD and RD groups and the additive deficits exhibited by the MDRD group suggest that an impairment to some other domain-general cognitive system mediates their ability to articulate definitions of common words.

The groups' performances on the Digit Span subtest followed a similar pattern to the one seen for the Vocabulary subtest. The MD and RD groups performed at a statistically-even level—both groups significantly lower than the typical achievement range—indicating that these participants experienced a specific deficit in verbal working memory. Just as in the Vocabulary subtest, the MDRD group performed at even lower level on average than the single-disability groups. In support of the additive hypothesis, the mean difference in scores between the TA and MDRD groups (3.57) was slightly more than the sum of the difference between the TA and MD groups (1.68) and the difference between the TA and RD groups (1.57); as described in section The Cognitive Profiles of Mathematical Disability, Reading Disability, and Comorbid Math and Reading Disability, this over-additivity was not the result of a significant interaction between math disability and reading disability, and therefore does not fit the synergistic hypothesis. These results suggest that individuals with math disabilities or reading disabilities alone present a specific, persistent impairment to their verbal working memory system, and that this impairment is even more pronounced in individuals with comorbid math and reading disabilities in a manner that is both statistically and clinically significant.

The verbal working memory deficit reported in the RD and MDRD groups was consistent with evidence from previous studies; individuals with reading disabilities consistently demonstrate impaired working memory (33, 81, 172, 173). The deficit demonstrated by the MD group, however, was not expected. Previous investigations about the relationship between impaired mathematical cognition and impaired working memory have yielded mixed results. In some studies, individuals with math disabilities exhibit deficits in working memory only when performing tasks that assess working memory and visuospatial reasoning simultaneously (68, 170); the Block Design subtest we used does not test these constructs simultaneously, because the prompt remains visible to the participant at all times (thereby remaining accessible in the participant's short-term sensory memory).

In other studies, individuals with math disabilities do not exhibit any significant deficits relative to controls when performing forward or backward recall tasks (174); but when wider selection criteria are used to classify participants into control and MD groups (i.e., below the 30th percentile on a standardized arithmetic test), the MD participants lag behind controls on memory span (175, 176). Given this contradicting evidence, we did not expect that the MD participants in the current study would demonstrate a specific working memory impairment; instead, the MD group did demonstrate pervasive difficulties in performing the working memory task.

Furthermore, the MDRD group—who was expected to exhibit a working memory deficit as seen in individuals with dyslexia—performed significantly poorer than both the MD and RD groups. Reminiscent of the pattern seen on the WAIS-R Vocabulary subtest, the difference in mean scaled scores between the TA and MDRD groups (2.21) was approximately even to the combined sum of the difference between the TA and MD groups (1.88) and the difference between the TA and RD groups (0.83). This provides further support to the additive hypothesis. An additive working memory deficit in MDRD participants has been previously reported in adults (74).

In contrast, Landerl et al. (90) reported that children with a math disability or reading disability alone did not exhibit significant deficits in working memory, but those with comorbid math and reading disabilities did—only on the backward-recall trials (and not the forward-recall trials) of the WISC-III Digit Span subtest. They found a significant interaction between math disability and reading disability on the comorbid group's mean scores on the backward Digit Span subtest, a result they interpreted as supporting the synergistic deficit hypothesis, but one that was not replicated in the current study. While it is unlikely that a deficit in working memory is the mechanism that underlies all forms of impaired numerical magnitude processing (67), again, the current results show that such is the case in linguistically-mediated math disability, since two separate, independent impairments to numerical cognition and reading ability contribute to a persistent, severe working memory deficit unique to individuals with comorbid math and reading disabilities.

### A Neuroeducational Model of Comorbid Mathematical and Reading Disabilities

**Figure 5:**
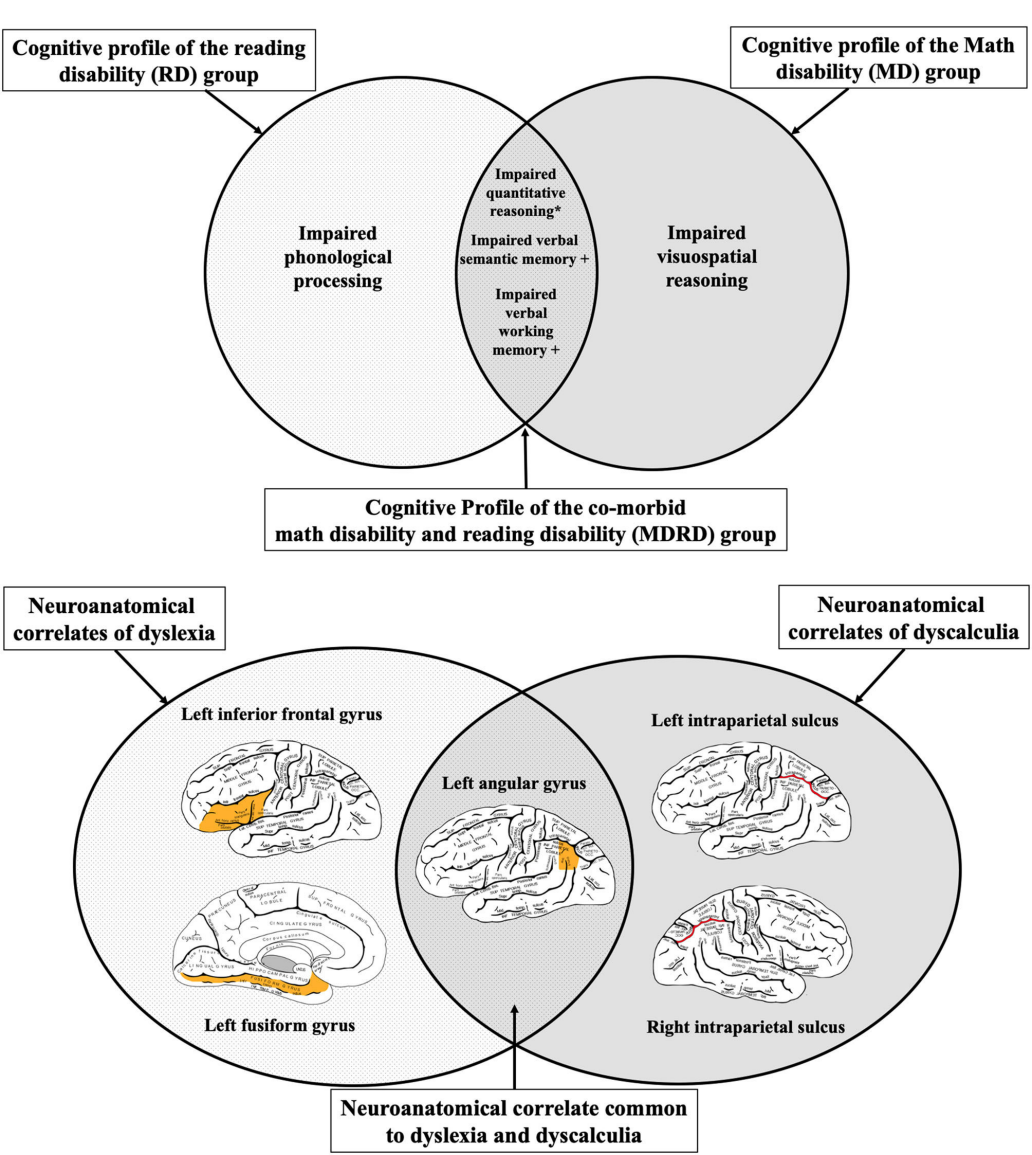
A neuroeducational hypothetical model of dyslexia-dyscalculia comorbidity. Top Panel: The neuroanatomical correlates of dyslexia and dyscalculia as determined by the systematic review. The left angular gyrus—a brain region where atypical physiological function has been reported in both individuals with dyslexia and individuals with dyscalculia. Bottom Panel: The cognitive profiles of individuals with reading disability (RD), math disability (MD), or comorbid math and reading disability (MDRD) in the current sample. The symbol “+” indicates an “additive deficit”, where the MDRD group demonstrated a significantly greater deficit than either the MD or RD group. The symbol “*” indicates an unexpected finding with novel implications for RD and MDRD; see Discussion. Brain diagrams were adapted from (45). Anatomy of the Human Body. Retrieved from https://www.bartleby.com/107/189.html.

The performance of the MDRD participants on the psychoeducational tasks in this study revealed two specific cognitive functions that are more severely impaired in comorbidity than in the single disability (see Bottom Panel of Figure 5). As expected, the MDRD group presented domain-specific deficits equal to those shown by the single-disability groups; their scores were on par with the RD group on two of the three phonological tasks, and also matched the performance of the MD group on the test of quantitative reasoning. More importantly, the MDRD participants exhibited domain-general deficits in verbal semantic memory and verbal working memory. In both the semantic memory task and the verbal working memory task, the magnitude of the MDRD group's deficit was approximately the sum of the separate deficits demonstrated by the MD and RD groups. These findings lend strong support to the additive deficit hypothesis of an independent math disability and an independent reading disability combined to produce a greater deficit in semantic memory and a larger deficit in working memory—deficits that were greater in magnitude than those observed in the participants with a single disability.

The model proposed in Figure 5 suggests that the left angular gyrus may be the key neurological structure that mediates the cognitive deficits uniquely expressed in both math and reading disabilities in both children and adults (see Top Panel of Figure 5). Previous studies have shown that atypical function of the left and angular gyrus is associated with deficits in phonological processing, numerical cognition, and working memory—all of which were exhibited by the MDRD participants in the current study.

Five strands of evidence form the basis for this hypothesis. First, functional neuroimaging studies have consistently identified the left angular gyrus as a pivotal structure in mediating word recognition, word decoding, and reading comprehension in normal readers (110, 177, 178). Individuals with reading exhibit less activation than normal readers at the left angular gyrus during tasks that involve effortful word decoding (such as the Word Attack subtest in this study), and also show decreased activation during semantic processing of visual and auditory words (179–181). In comparison to controls, individuals with dyslexia show atypical hypoactivity at the left angular gyrus while performing phonological tasks (55, 56) as well in individuals with dyscalculia while performing tasks that test the approximate number system (84, 182). Second, lesions to the posterior cerebral artery (the blood supply of the left angular gyrus) produces symptoms of alexia (157), while lesions to the gyrus itself can produce acquired dyscalculia, alongside other symptoms of Gerstmann's syndrome (183).

Third, the left angular gyrus has been shown to mediate the two aspects of memory retrieval: the retrieval of phonetic representations of familiar words (138) and the retrieval of arithmetic facts (85). The latter demonstrates a contrast in the type of calculations which involve differentially the right and left hemisphere; the right intraparietal sulcus is associated with greater activation for processing inexact quantities for calculations involving the approximate number system, while the left angular gyrus and left intraparietal sulcus are involved in retrieving exact quantities for calculations that require a single exact solution. In addition, the left angular gyrus has shown low functional connectivity with the inferior frontal and left fusiform gyri during phonological processing tasks (177, 184).

Fourth, impairments to subcomponents of working memory can be mapped to the disruption frontal-lobe-to-parietal-lobe association fibers, converging on the left angular gyrus. According to Baddeley's model of Working Memory (185), a subcomponent of working memory called the phonological loop facilitates the short-term storage encoding verbal information into long-term memory. In Positron Emission Tomography (PET) studies with normal readers performing pseudowords tasks similar to the psychoeducational tests used here, researchers have shown that the angular gyrus is directly active in facilitating the short-term storage and retrieval of unfamiliar phonemic sequences (173, 186).

Lastly, applying TMS (Transcranial Magnetic Stimulation) to the left angular gyrus has been shown to increase the accuracy of semantic memory when pairing stimuli for classical conditioning (187), but can also cause deficits in visuospatial reasoning (mainly right-left disorientation). This collection of evidence from functional imaging, lesion-symptom analysis, and neuropsychology research provide valuable insight into the unique role of the left angular gyrus in mediating the cognitive deficits shared in math and reading disabilities.

The MDRD participants demonstrated additive domain-general deficits in verbal working memory and verbal semantic memory, which suggests a functional relationship between the verbal, expressive component of these deficits, and the retrieval of symbolic and semantic representations of words from long-term memory. A possible explanation is that a pervasive deficit to the verbal working memory system (specifically the phonological loop subcomponent in Baddeley's model) impairs both the short-term storage of basic verbal information and the retrieval of semantic information from long-term storage. The model shown in the Bottom Panel of Figure 5 proposes that a pervasive deficit in the temporary storage of verbal information is an additional core deficit unique to individuals with comorbid dyslexia-dyscalculia, associated with atypical function of the left angular gyrus.

## Conclusions

In summary, converging evidence from neuroimaging and psychoeducational research suggests that impaired phonological, numerical, semantic, and working memory processes may be related to dysfunction of the left angular gyrus in individuals with dyslexia and individuals with dyscalculia. Individuals with dyslexia exhibit atypical hypoactivation at the inferior frontal gyrus when performing the same psychoeducational tests that are used to assess phonological processing deficits in individuals with reading disabilities. Individuals with dyslexia also exhibit atypical hypoactivation at the visual word form area of the left fusiform gyrus (located on the sagittal surface of the temporal lobe) when viewing written words, and consistently demonstrate impaired word recognition. These cognitive profiles are similar to those seen in the present study, where individuals with reading disabilities demonstrated significant deficits on tests of vocabulary and reading fluency. Similarly, individuals with dyscalculia exhibit atypical hypoactivation of the bilateral intraparietal sulci when performing numerical magnitude processing tasks, akin to the psychoeducational tests used to assess the function of the approximate number system in individuals with mathematical disabilities.

Multiple sources of evidence show that individuals with dyslexia and individuals with dyscalculia both exhibit dysfunction of the left angular gyrus. The left angular gyrus is the only neuroanatomical region where abnormalities in physiological activation, white matter volume, and white matter tractography have been reported among individuals with dyslexia and individuals with dyscalculia in functional neuroimaging, structural neuroimaging, and functional connectivity studies. Individuals with dyslexia exhibit atypical hypoactivation at the left angular gyrus when performing word definition recall tasks, demonstrating the same deficits in semantic memory that are seen in individuals with reading disabilities when performing the WAIS-R Vocabulary task. Individuals with dyscalculia exhibit atypical hyperactivation at the left angular gyrus when retrieving arithmetic facts, presenting similar difficulties as individuals with math disabilities when performing psychoeducational tests that assess basic math fluency. Furthermore, the left angular gyrus is the only neuroanatomical region where lesions have been identified in individuals with alexia as well as individuals with acalculia. The individuation of such area as the main overlapping brain structure associated with reading-math comorbidity makes sense anatomically since meta-analytic evidence suggests its role is to specifically associating language with other types of information and could be regarded as a language processing marginal area participating in an “extended Wernicke's area” or “Wernicke's system” (188).

Based on past and present evidence, we conclude that children and adults with comorbid math and reading disabilities demonstrate domain-specific deficits equivalent to single-disability individuals. However, individuals with comorbidity also demonstrate additive domain-general deficits in verbal working memory and verbal semantic memory. According to the evidence gained from the exhaustive review of the literature, the domain-specific reading deficits may correspond to developmental differences in the left inferior frontal and fusiform gyri, and mathematical deficits can be traced to developmental differences in the bilateral intraparietal sulci. The current model proposes that domain-general, additive deficits in semantic memory and verbal working memory—two pervasive impairments that are unique to neuropsychological profile of individuals with comorbid math and reading difficulties—may be the result of atypical development of the left angular gyrus.

Looking ahead, most recent advances in the emerging field of neuroimaging genetics have revealed bilateral interplay between genetic profile and neurological function and sometimes even a trilateral interaction between the latter and neuroanatomy. These studies have primarily focused on dyslexia (189–192), but similar evidence is also emerging for dyscalculia (193–195) and first evidence has appeared for their comorbidity (132). Although exhaustive review is beyond the scope and space of this paper (for very recent reviews see (193, 196), our model and research synthesis is complementary and could be further probed with neuroimaging genetics, specifically, in the pursuit of precise early detection of comorbid reading and math disability well before they emerge in formal educational settings. While genetic profile is insufficient to fully explain a complex condition such as dyslexia-dyscalculia comorbidity, some genetic constellations, or endophenotypes, can clearly help in early diagnosis (197). Because early intervention might be crucial to address this complex severe condition, genetics might be an instrument helping to close or at least narrow the diagnostic gap in the early years of life, before schooling. The present synthesis and model could be a starting point for the timely identification of reliable endophenotypes linked with early development of the angular gyrus and later psychoeducational diagnosis of reading-math disability comorbidity.

## Data Availability Statement

The datasets generated for this study are available upon reasonable request in a form being coherent to all institutional, national and international ethical standards (e.g., anonymization), including intellectual property guidelines.

## Ethics Statement

The studies involving human participants were reviewed and approved by Research Ethics Board of The University of British Columbia. Written informed consent to participate in this study was provided by the participants' legal guardian/next of kin.

## Author Contributions

JG curated database, designed and conducted main analyses, and wrote the basis of the first draft. LS designed and led the prospective cohort study and database, obtained funding, and revised draft. AD'A collected data, helped design the study, helped with database curation and management, helped with data analysis, obtained further funding, oversaw writing of the final manuscript from first to final draft, and edited into final manuscript. All authors contributed to the article and approved the submitted version.

## Conflict of Interest

The authors declare that the research was conducted in the absence of any commercial or financial relationships that could be construed as a potential conflict of interest.
